# Green Nano-Biotechnology: A New Sustainable Paradigm to Control Dengue Infection

**DOI:** 10.1155/2022/3994340

**Published:** 2022-08-08

**Authors:** Tanzeel Zohra, Ali Talha Khalil, Faryal Saeed, Bushra Latif, Muhammad Salman, Aamer Ikram, Muhammad Ayaz, H. C. Ananda Murthy

**Affiliations:** ^1^Public Health Laboratories Division, National Institute of Health, Islamabad, Pakistan; ^2^Department of Pathology, Lady Reading Hospital Medical Teaching Institution, Peshawar, KP, Pakistan; ^3^Department of Biotechnology, Quaid-i-Azam University, Islamabad, Pakistan; ^4^Department of Pharmacy, Faculty of Biological Sciences, University of Malakand, Chakdara, KP 18000, Pakistan; ^5^Department of Applied Chemistry, School of Applied Natural Science, Adama Science and Technology University, P.O. Box 1888, Adama, Ethiopia

## Abstract

Dengue is a growing mosquito-borne viral disease prevalent in 128 countries, while 3.9 billion people are at high risk of acquiring the infection. With no specific treatment available, the only way to mitigate the risk of dengue infection is through controlling of vector, i.e., *Aedes aegypti*. Nanotechnology-based prevention strategies like biopesticides with nanoformulation are now getting popular for preventing dengue fever. Metal nanoparticles (NPs) synthesized by an eco-friendly process, through extracts of medicinal plants have indicated potential anti-dengue applications. Green synthesis of metal NPs is simple, cost-effective, and devoid of hazardous wastes. The recent progress in the phyto-synthesized multifunctional metal NPs for anti-dengue applications has encouraged us to review the available literature and mechanistic aspects of the dengue control using green-synthesized NPs. Furthermore, the molecular bases of the viral inhibition through NPs and the nontarget impacts or hazards with reference to the environmental integrity are discussed in depth. Till date, major focus has been on green synthesis of silver and gold NPs, which need further extension to other innovative composite nanomaterials. Further detailed mechanistic studies are required to critically evaluate the mechanistic insights during the synthesis of the biogenic NPs. Likewise, detailed analysis of the toxicological aspects of NPs and their long-term impact in the environment should be critically assessed.

## 1. Introduction

In the age of emerging and reemerging pathogens, resistant bugs, deadly cancers, and neglected tropical diseases like dengue necessitate the need of holistic approaches to foster health and well-being [1–4]. In this regard, the mosquito-borne diseases got immense significance as mosquitoes serve as a vector for various deadly infections like yellow fever, malaria, filariasis, dengue, etc. [5]. Among the mosquito-borne viral diseases, dengue fever has attracted attention of researchers, epidemiologists, health, and social workers [6], because of their life threatening nature, massive disease burden, climatic conditions, vector expansion, urbanization, and other socio-demographic factors [7]. The dengue virus is transmitted by the *Aedes aegypti*, and *Aedes albopictus* has put billions of people at risk of the dengue infection, especially threatening the tropical and subtropical regions [8, 9]. The annual reported cases of the infection are estimated to be between 50 to 100 million. It is further estimated that the actual number of the dengue incidence are around 390 million with 96 million of symptomatic cases and 25,000 estimated annual mortalities” [10]. Dengue has now an endemic status in 128 countries. The situation is further aggravated by the resistant strains of dengue which are proposed to be the primary cause of the transmission on a large scale. The origination of resistant strains of dengue virus is the main cause of dissemination of dengue infections and its influence on human health. Dengue virus has four different serotypes, referred as DENV 1–4, that have substantial genotypic variations within each serotype. Recently, the fifth serotype of the dengue virus (DENV-5) was also identified [11]. Infection caused by all serotypes reveals similar symptoms [12]. Lifelong immunity is achieved upon recovery of the patient from one particular serotype, while the recovered patient is not protected from a secondary infection from other serotypes. The secondary infection may lead to more severe cases like dengue shock syndrome (DSS) and dengue hemorrhagic fever (DHF) [13]. DSS and DHF results through the antibody mediated disease enhancement (ADM), resulting in either from the previous infection or induced by the vaccine [14]. Dengue infection has no specific treatment, while the only option is supportive care and symptomatic treatment. Therefore, an early diagnosis and vector management is a key to controlling dengue fever.

As of now, despite tremendous research for antiviral drugs or moieties, there has been no significant development to combat the DENV, and usually, symptomatic treatment is provided to the affected patients. At present, the WHO recommends only one dengue vaccine for all serotypes in children >9 years [15, 16]. The vaccine is only implemented in countries with greater than 70% sero-prevalence of the dengue virus; however, the vaccinations are only recommended for dengue sero-positive cases [17]. Extensive research is required to develop synthesize chemical entities that enable to inhibit the virus. E-gene, NS-1 gene, and NS-3 genes are considered as potential pharmaceutical targets for drugs. Previous studies revealed that bromocriptine exhibit antiviral potentials by inhibiting its replication. Other drugs like balapiravir, chloroquine, prednisolone, and celgosivir have not revealed any significant results during trials. Clinical trials with other drugs like ribavirin, ketotifen, and ivermectin are currently underway. Other researchers have been tirelessly working to search anti-dengue phytochemicals that can be useful in the control of dengue. The prevalence of dengue fever has prompted scientists to look for novel therapies, antiviral drugs, and nanotechnology based innovations. This study aims to update researchers' knowledge about the use of natural products-mediated synthesis of biogenic NPs and their possible role in the management of dengue infection and anti-dengue mechanisms of biogenic NPs.

## 2. Mitigating the Dengue Infection

Dengue virus represents Flaviviridae having a spherical shape and size of ∼50 nm [18]. Dengue virus comprises ten proteins, in which 3 are structural proteins and 7 nonstructural proteins (NS). These nonstructural proteins play an important part in immune evasion, replication, and assembly of the virus. Nonstructural proteins like NS-1, NS-3, and NS-5 are absolutely vital for formation of viral particles and, therefore, also present an opportunity to design effective antiviral drugs. Dengue prevalence is a pressing problem for the developing world that signifies a dire need of innovative approaches for curing the disease or limiting their prevalence. There is a need for novel anti-dengue agents apart from transcription or protease activity that works on viral stages. Entry inhibitors alongside fusion are viable options that limit dengue entry into the target cell, repressing its replication and rendering the virus ineffective [19, 20].

Currently available vector control strategies are grouped into including physical control via GIS mapping for locating dengue foci, effective surveillance, determination of oviposition sites, and community-driven control programs. Next strategy is through biological control including paratransgenesis, vectors genetic modifications, sterile insects techniques, and use of crustacean and larvivorous fish, whereas chemical control strategies include the use of insecticides, plant derived compounds, use of insects growth regulators, and the “attract and kill” approach using pheromones. Others include immunotherapy strategies via the use of vaccines. Such approaches encompass biological, chemical, and environmental methods to curtail breeding and proliferation of the vector for dengue virus, i.e., *Aedes aegypti.* Due to the lack of awareness, poor sanitation hygiene, and other socio-economic motives, vector control becomes more challenging in developing countries [21, 22]. Effective and efficient vector control strategies through chemical or biological products are used worldwide [23]. However, chemicals such as synthetic lead have powerful impacts on public health that bring about resistance in different species of mosquitoes [24, 25]. Eco-friendly ways to control mosquito vectors with ultra-efficiency are needed. The mosquito is generally targeted by organophosphates and other growth regulators. Indoor spraying and bed nets are used to decrease the transmission. Phytochemicals with strong mosquitocidal and insecticidal potential are considered an alternative to synthetic insecticides in vector control programs. These plant-derived bioactive entities are characterized by their larvicidal, pupicidal, and adulticidal properties. Furthermore, both naturally occurring and synthetic chemicals are revealed to alter the oviposition behavior in mosquitoes or possess the ovicidal properties or may act as mosquito repellant [19, 26–29].

Scientists have also proposed certain genetic strategies to prevent the transmission of DENV to human beings. This is done by the introduction of the genes responsible for the disease resistance in the vector. Among them, one of the endosymbiotic bacteria (*Wolbachia*) is frequently used to spread disease resistant genes into mosquitoes. A transfected line of the *Aedes aegypti* with *Wolbachia* revealed suppression of the DENV by increasing the basal immunity in the insect that led to the reduced transmission. These *Wolbachia* transfected *A. aegypti* female mosquitoes possess an additional reproduction advantage over the uninfected ones [30]. Other researchers have tried to use the life span shortening strain of *Wolbachia,* to reduce the lifespan of the mosquito, which can decrease the burden of the vector borne diseases spread by *A. aegypti* [31]. Such genetic strategies are, however, primitive and mostly successful at the lab scale, while their implementation on ground would require a deeper understanding of the underlying mechanisms and further research.

## 3. Nano-Biotechnology, an Emerging Interface

The successful apprehension and manipulation of nanomaterials using the environmentally benign resources like plant extracts or their derived chemical entities have paved a way for using nanotechnology in an economical, sustainable, and compatible way [32–34]. The process is characterized by treating plant extracts with metal salts in different combinations that lead to the reduction of metal salt and subsequent capping and stabilization of NPs [35, 36]. The convergence of nanotechnology and biotechnology has revealed exciting results for different health-hygiene, nanomedicinal, environmental, and industrial applications [37–39]. These applications have paved a way for the crystallization of nano-biotechnology or nanobiology. Metal NPs like silver, gold, zinc, etc., are known to possess multifunctional properties owing to their unique surface area to a volume ratio. These NPs can be assembled by a variety of physical, chemical, or biological processes [40, 41]. The physical means are often characterized by high energy inputs making the overall process expensive while chemical means can generate hazardous wastes [42].

Recently, medicinal plants have been reported to exhibit efficacy in various diseases including cancer, infectious diseases, diabetes, and neurological disorders [43–50]. They inhibit the dengue virus by blocking the replication of virus particles through interacting with the genome, or by blocking their entry. The anti-dengue effect is manifested through destabilization of NS proteins. Natural products obtained from plants are reported to stop the viral replication either by interfering with the enzymes like inhibiting polymerases, interacting with glycoproteins, or inhibiting the replication by interfering with the RNA synthesis pathway. Despite the advances in screening potential inhibitors, no such therapies have been approved due to the heterotypic dengue infections [51–55].

A significant volume of research is now focused on the biological methods that include extracts from the medicinal plants as an eco-friendly, simple, and economical process for assembling nanomaterials or composite nanomaterials [56–61]. Other biological forms like microorganisms can also be utilized for the synthesis of metal NPs [33] but possess additional requirements like culture maintenance and sterile conditions. On the contrary, plants do not possess any expensive requirements, and are easy to handle. Phytochemicals can reduce and stabilize NPs [62]. Apart from the industrial applications, these biogenic NPs have revealed excellent biomedical potential [63, 64]. Converging experimental evidence suggests that the biogenic NPs can be used against the dengue virus and controlling their vectors [19]. The phyto-fabricated NPs present an excellent opportunity to control the dengue virus. A detailed review of the literature is presented in Table 1, summarizing the plant used, type of the metal NPs, their size, and application in vector control.

## 4. Anti-Dengue Properties of Biogenic Nanoparticles; Molecular Aspects

**Figure 1:**
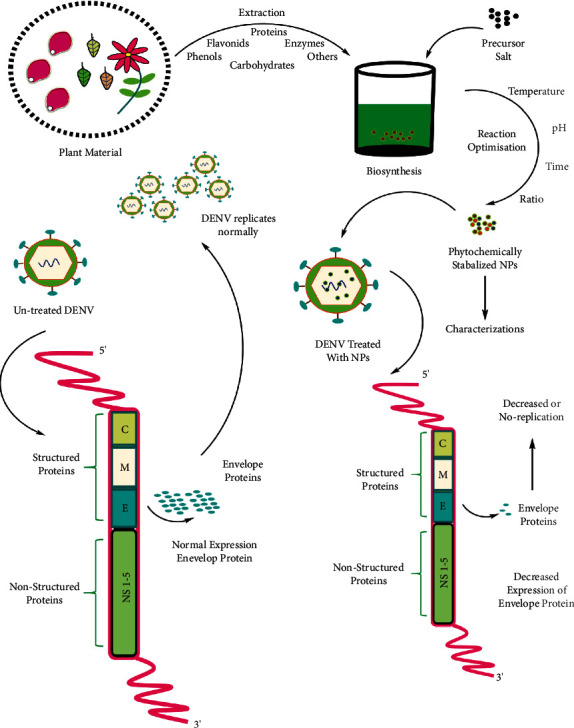
Molecular interaction of biogenic NPs with the DENV genome causing decreased expression of viral E-gene.

Few studies have documented the anti-dengue effect of the phytogenic silver NPs against DENV-2. The likelihood utilizing green-synthesized NPs in the fight against dengue (serotype DEN-2) has been acknowledged lately. One of the research articles encompasses the biosynthesis of silver NPs from *Bruguiera cylindrica* (L.) Blume and evaluated their effects on the dengue virus as well as their toxicity was evaluated against the vector [65]. Interestingly, the silver NPs treatment revealed decreased expression of dengue viral E-gene that codes for structural envelope (*E*) protein. These results were confirmed through the western blot and RT-PCR. The viral E-gene was found to be down-regulated in a dose dependent manner leading to significant reduction in envelope proteins as compared to the control. Significant downregulation at 30 *µ*g·mL^−1^ was observed. The synthesized silver NPs were found to be toxic to the *A. aegypti* larvae and pupae. Similar results are concluded for the *Moringa oleifera* synthesized silver NPs for anti-dengue applications [19]. Sonneratia alba Sm. derived silver NPs tested in the concentration range of 5 *µ*g/mL to 15 *µ*g·mL^−1^ also revealed significant reduction in the Viral E-protein, indicating a potential anti-dengue effect [66]. The aforementioned findings put forth the hypothesis that the reduction in the formation of *E* protein may occur due to silver NPs inhibiting the *E* gene and reducing the number of units that are ineffective [65]. Subsequently, *Centroceras clavulatum* (C.Agardh) Montagne synthesized silver nanoparticles (AgNPs) that were tested at 50 mg/ml showed no toxicity which is relevant against Vero cells, while the inhibition of growth of DEN-2 viral occurred for more than 80 percent [67]. The importance of screening different biosynthetic methods has been felt by these studies that can explore ways for the production of novel and safer nano drugs producing NPs having different features. Available studies show the important role of screening different plants which act as a source of reducing molecules of nanosynthesis because different paths frequently guide us to manifold various aspects of NPs and characteristics of biological toxicity [66] (Figure 1).

Conclusively, these studies show strong and tangible potential of screening substantial species of plants for biosynthesis of NPs with anti-dengue applications. The scarce literature further necessitates conducting assemble NPs other than silver, using medicinal plants for investigating their anti-dengue properties.

### 4.1. Phyto-Nano-Interface for Vector Control

The use of synthetic insecticides for potential vector control is undesired because of environmental hazards and the elimination of the nontarget organisms [68, 69]. Besides, environmental issues, health concerns, and emerging insect resistance to insecticides have led to the realization that these synthetic chemicals may not be reliable in the long-term [70]. Such pesticides are an instant danger to human health if used in a nonjudicious manner. According to estimates, the synthetic pesticides lead to around 3 million cases of poisoning and 222,000 deaths annually. Similarly, escaping of the pesticides residues and their accumulation in the food chain represents an unforeseen danger [71]. Thankfully, nanotechnology-based interventions have emerged has a promising and alternative source of insecticides due to their potent insecticidal nature, mobility, solubility, and stability [70]. The promising potential of green-synthesized NPs has paved a way for novel vector control strategies. Their toxicity against some arthropod pests and vectors, especially mosquitoes has been well documented. There is a significant volume of literature on the toxicity of biogenic NPs on mosquitoes; however, the information on the precise mechanistic aspects is scarce. The underlying mechanism is pivotal to investigate the toxicological consequences arising from the use of NPs as pesticides.

**Figure 2:**
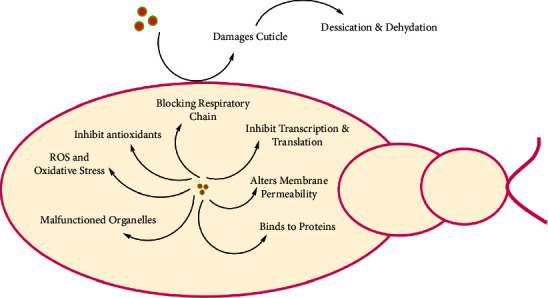
Mechanism of nanoparticles toxicity against insects.

The toxic effect of NPs may be linked to some stress stimuli caused by NPs (Figure 2). The exact mechanism is not understood completely but scientific findings have revealed that NPs may cause morphological alterations like loss of lateral hair and damaged gills and brushes [72]. This may affect the respiratory activity of larvae, since the larval stages rely solely on gills for breathing. At the cellular level, severe membrane degradation is observed, as NPs penetrate easily through the membrane. NPs may get accumulated in midgut causing shrinkage of abdomen and damaged epithelium or cortex. Blocking of the trypsin enzyme activity is also considered as one of the causes of NPs mediated insecticidal activity [73]. Activity of this digestive protease is linked with the signal transduction system as it regulates the expression of a second gene, i.e., the late trypsin gene. The presence of two trypsin allows the mosquito to assess the quality of the meal and adjust the levels of late trypsin for a particular meal with remarkable flexibility. Feeding activity is disturbed when trypsin activation is halted and the quality of meal cannot be assessed [74]. Another factor contributing to the toxicity of NPs is directly related to their small size due to which they can pass easily into the cuticle and act directly on epidermal cells and interfere with enzyme production necessary for tanning and cuticle oxidation, ultimately affecting the whole molting process. Alternatively, they may inhibit neurosecretory cells resulting in cuticular shrinkage. Some NPs are also associated with the disturbing of muscular layers causing loss of distinction in endocuticle and exocuticle leading to insect inactivity. NPs may bind to the cuticle, sorbing the cuticular lipids and waxes resulting in body wall desiccation, de-pigmentation, abrasion, spiracle blockage, and insect dehydration, to which the insect ultimately succumbs [72, 74]. This factor contributes to the utilization of NPs against the early instars and pupae and prevents their development to adult stage rendering them as a powerful larvicidal agent [75]. Authors have reported interruption of acetylcholinesterase activity by NPs. Acetylcholine is a compound involved with nerve impulse transmission from nerve to nerve cell or involuntary muscles, and this activity is regulated by acetylcholinesterase (AChE) [63, 76]. It is reported the NPs interfere with AChE resulting in disturbance of nerve impulse transmission across cholinergic synapses [77]. Therefore, this could be useful to assess the potential neurotoxic capacity of some NPs [74]. Hormonal imbalances are also reported in insects which are manifested by NPs. Further, NPs are reported to interfere with the cytochrome P450, involved in the molting of insects [73, 78]. A critical impact on reproduction and development is also reported [74], where Gonadotropin production is downregulated resulting in reduced fitness and reproductive failure. Reduced female fertility is observed as NPs disrupt the oogenesis process and ovaries become defective, having a negative effect on egg laying capabilities [72]. Moreover, NPs damage the organism by penetrating through the exoskeleton [79], enter in the intracellular space, and then the nanoscale material binds to sulfur from proteins or to phosphorus from DNA which leads to the rapid denaturation of organelles and enzymes. Due to the decrease in membrane permeability and disturbance in proton motive force, loss of cellular function, and cell death occur [80, 81]. At the cellular level, NPs can penetrate the cytosol and interrupt the cellular signaling pathways, causing disruption in ion exchange and neuromuscular coordination [73].

**Figure 3:**
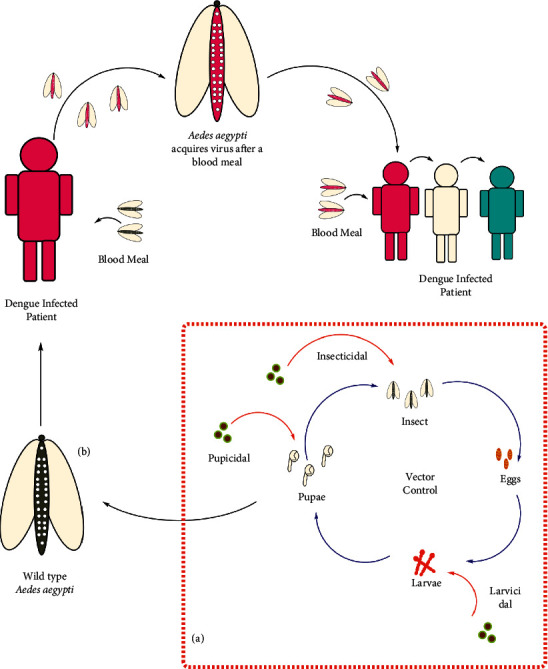
Vector control and dengue transmission.

Even though several evidences exist on the toxicity of NPs, different experimental designs with diverse NPs sizes, coatings, concentrations, times of exposure, measured endpoints, and cell types make it difficult to compare results and determine the mode of action by which these particles inflict damage to organisms [82–84]. Generation of reactive oxygen species (ROS) and free radicals have been observed and implicated in the cause of oxidative stress, namely, in the form of antioxidant defense system activation/inhibition such as depletion of glutathione, lipid peroxidation and DNA damage, decreased mitochondrial activity, inflammatory processes, and apoptosis in a wide variety of cell types [85] (Figure 3).

Converging evidence suggests an inverse correlation between the size of NP and their toxicity and penetration into the body of insects. Despite a number of pieces of evidences, there is a dire need to conduct extensive studies on the effects of the biogenic metal NPs on insects with reference to their physicochemical nature like size, shape, charge, etc. Moreover, the present body of literature only indicates silver and gold NPs for their anti-parasitic properties and applications in entomology. Research can be extended to other metal NPs of composite nonmaterial's biosynthesized from medicinal plants.

NPs: nanoparticles; X-ray diffraction (XRD); Fourier transform infrared (FTIR); scanning electron microscope (SEM); energy dispersive X-ray analysis (EDX); UV-visible spectroscopy (UV-vis); field emission scanning electron microscope (FESEM); high resolution transmission electron microscopy (HRTEM); transmission electron microscopy (TEM); dynamic light scattering (DLS).

## 5. Nanoparticles Enhances Predation Efficiency

Biological control of dengue vectors seems another probable solution. The prospective biological control of dengue vectors can be performed using natural predators like fish, young instar tadpoles, copepods, and water bugs. Fishes were predominantly considered for biological control of mosquitoes. Places that have the possibility to breed mosquitoes such as dams, marshes, canals, ponds, etc., were inundated with numerous predatory fishes [148]. The cyclopoids are also reported to be among the efficient predators of the larvae of the mosquito involved in the spread of dengue [113]. Copepods represent another economical and cost-effective biological control of culicidae larvae in urban and semiurban areas [166, 167]. The most effective agents of copepods that control mosquitoes biologically are *Mesocyclops*, i.e., *Mesocyclops pericornis, Mesocyclops longisetus, Mesocyclops guangxiensis, and Mesocyclops thermocyclopoides* [113]. Recently, the effect of NPs on the predation behavior of these natural predators has been studied (Table 2). The striking findings are the increase in predation efficiency. It has been clearly demonstrated that the rate of predatory activity rises up administering NPs; however, the underlying exact mechanism is yet to be explored. The efforts, however, have been made to investigate the nontarget effects of NPs towards predatory copepods are somewhat limited.

## 6. Conclusion and Insights for Future Research

In the synthesis of the metal nanoparticles, the green synthesis method stands out due to its eco-friendly and sustainable nature. Based on the available research, it can be concluded that the biogenic nanoparticles have an enormous potential to answer the pressing healthcare challenges, such as the mitigation of the dengue infections. Dengue virus is now considered as global threat that requires innovative approaches for its control. Nano-biotechnology interventions can be helpful in reducing the disease burden in a cost-effective and sustainable manner. Biogenic nanoparticles can reduce the dengue infection with by direct interaction or indirect interaction with the vector. Numerous studies have supported the potential of biogenic NPs for manifesting the anti-dengue effect by interfering and downregulating the critical structural genes necessary for the viral assembly. Furthermore, these biogenic NPs have successfully demonstrated vector control potential which is manifested through their biocidal nature. From an application standpoint, the production of these biogenic NPs is free of any hazardous chemicals, with no special energy requirements and an easy scale up potential. The challenge is to implement these nano-biotechnology-based interventions on ground.

The major focus in the green synthesis is centered on the synthesis of silver and gold nanoparticles; however, these studies should be extended to other innovative composite nanomaterials. Literature of the mechanistic insights of green synthesis is scarce and further studies should be undertaken to critically evaluate the mechanistic insights during synthesis of the biogenic nanoparticles. Similarly, detailed studies should be conducted to evaluate the toxicity of the nanoparticles and their long-term impact in the environment should be critically assessed.

## Figures and Tables

**Table 1:** Plant based nanoparticles used against the dengue vector.

Sr. No	Plant used	Target Stage	Mechanism	Type of NPs	Characterization			MIC	References
					Size	Shape	Technique used		
1.	*Leucas aspera* (willd.) link	Larvae	Not reported	AgNPs	25–80 nm	Clustered and irregular shapes, and mostly aggregated	UV-vis, XRD, FTIR, SEM	0.01–5 mg·L^−1^	[86]
2.	*Feronia elephantum* L.	Larvae	Not reported	AgNPs	20 to 60 nm	Triangular, pentagonal, and hexagonal structures	UV-vis, FTIR, SEM, EDX,TEM	37.534 *μ*g·mL^−1^	[87]
3.	*Annona muricata*	Larvae	Not reported	AgNPs	20 to 53 nm	Spherical	UV-vis, FTIR, XRD, SEM, EDX, TEM	—	[88]
4.	*Phyllanthus niruri* L.	Larvae	Larvae is perforated through the breathing tube, eradicating them by contamination and suffocation	AgNPs	30–60 nm	Spherical, mostly aggregated	UV-vis, SEM, EDS, FTIR, XRD, EDX	—	[89]
5.	*Holarrhena antidysenterica*	Larvae	Not reported	AgNPs	20 to 80 nm	Dispersed, crystalline, and mostly spherical	UV-vis, XRD, SEM, TEM, FTIR	—	[90]
6.	*Coleus aromaticus* Lour.	Larvae	Not reported	AgNPs	262.7 to 553.9 nm	Spherical and aggregate	UV-vis, EDX, FTIR, XRD, SEM	—	[91]
7.	*Artemisia vulgaris*	Larvae	Interfere with molting and other physiological processes	AgNPs	30–70 nm	Polydispersed, irregularly shaped	UV-vis, FTIR, XRD, EDX, SEM	—	[92]
8.	*Gracilaria firma*	Larvae	Not reported	AgNPs	12–200 nm	Spherical	UV-vis, FTIR, XRD, EDX, TEM	—	[93]
9.	*Myristica fragrans*	Larvae	Not reported	ZnO NPs	100 to 200 nm	Rod-like	UV-vis, DLS, FTIR, Zeta Potential, XRD.EDX, SEM, TEM	—	[94]
10.	*Beauveria bassiana*	Larvae	Not reported	AgNPs	36.88 to 60.93 nm	Spherical	UV-vis, SEM, EDX	—	[95]
11.	*Aganosma cymose*	Larvae	Not reported	AgNPs	1 to 16.5 nm	Polydispersed, spherical	UV-vis, FTIR, XRD, AFM, SEM, TEM, XRD, AFM	—	[96]
12.	*Cocos nucifera*	Larvae	Inhibition of major detoxifying proteins glutathione-S-transferase and cytochromeP450	AgNPs	5–65 nm	Spherical, pseudo spherical and rectangle	UV-vis, TEM, XRD	—	[97]
13.	*Carissa carandas*	Larvae	Route through the exoskeleton of insect into cells of individual and intervention with sloughing	AgNPs	1.6 to 7.4 nm	Spherical poly-dispersed	UV-vis, FTIR, XRD, AFM, SEM, TEM	—	[98]
14.	*Zeuxine gracilis*	Larvae	Not reported	AgNPs	20–40 nm	Orbicular, cubic	UV-vis, EDX, FTIR, XRD, DLS, SEM,TEM	—	[99]
15.	*Halodule uninervis*	Deformed adults	Inhibit neurosecretory cells, shrink internal cuticle, and/or can act directly on epidermal cells causing cuticular oxidation	AgNPs	25–40 nm	Spherical or with cubic	UV-vis, FTIR, SEM, EDX, XRD, Raman analysis	—	[100]
16.	*Chomelia asiatica*	Larvae	Not reported	AgNPs	15–31 nm	Triangular, pentagonal, and hexagonal	UV-vis, FTIR, SEM, EDX	—	[21]
17.	*Parthenium hysterophorus*	Larvae	Not reported	TiO_2_ NPs	20–50 nm	Spherical	UV-vis, FTIR,SEM, EDX, XRD	—	[101]
18.	*Sida acuta*	Larvae	Not reported	AgNPs	20 to 60 nm	Spherical, triangular, pentagona l, and hexagonal	UV-vis, FTIR, SEM.TEM, EDX	—	[102]
19.	*Arachis hypogaea*	Anal papillae region and cuticle layer.	Reduce membrane permeability, deactivate enzymes in midgut, liberate peroxides leading to cell death	AgNPs	20 to 50 nm	Spherical and polyhedral	FTIR, XRD, TEM, SEM, EDX	—	[103]
20.	*Azadirachta indica*	Larvae and pupae	Penetration through the membrane	AgNP	30 to 50 nm	Spherical	UV-vis, FTIR, SEM, EDX, XRD	3.969 (larva I) to 8.308 ppm (pupa)	[104]
21.	*Heliotropium indicum*	Larvae	Not reported	AgNP	18 to 45 nm	Spherical, triangle, truncated triangles, and decahedral	UV-vis, FTIR, TEM, SEM, EDX, XRD	35.97 *μ*g/mL	[105]
22.	*Feronia elephantum*	Larvae III	Bind to sulfur-containing proteins or phosphorus- containing compounds like DNA, causes denaturation of some enzymes and organelles	AgNP	20 to 60 nm	Triangular, pentagonal, and hexagonal	UV-vis, FTIR, SEM, EDX, XRD	23.12 *μ*g mL^−1^	[106]
23.	*Carmona retusa*	Larvae	Not reported	AgNP	20 to 40 nm	Cubic	UV-vis, XRD, FTIR, TEM, SAED	198.766 ppm	[107]
24.	*Plumeria rubra*	Larvae II, IV	Not reported	AgNP	32–200 nm	Spherical	UV-vis, TEM, PSA and zeta potential	500 ppm	[108]
25.	*Catharanthus roseus*	Larvae	Altered physiological processes	AgNP	35 to 55 nm	Spherical	UV-vis, H1NMR, FTIR, and mass spectroscopy	40 ppm	[109]
26.	*Anisomeles indica*	Larvae III	Nor reported	AgNP	18 and 35 nm	Spherical	UV-vis, FTIR, SEM, EDX	35.21 mg/mL	[110]
27.	*Ulva lactuca*	Larvae IV	Gastric caeca, muscles, nerve cord ganglia appeared damaged and disorganized, spoiled epithelium	ZnO NPs	10–50 nm	Sponge-like asymmetrical	XRD, UV-vis, FTIR, SAED, TEM	50 *μ*g/ml	[111]
28.	*Sargassum muticum*	Larvae and pupae	Binds to sulfur from proteins or to phosphorus from DNA, causes swift denaturation of organelles and enzymes	AgNP	43–79 nm	Spherical	FTIR, SEM, EDX, and XRD analyses	10 ppm	[112]
29.	*Cymbopogon citratus*	Larvae and pupae	Interfere with molting and other physiological processes	AuNPs	20–50 nm	Orbicular, trigonal, hexagonal, and rod- like	UV-vis, FTIR, TEM, EDX, XRD	41.5 ppm	[113]
30.	*Pedilanthus tithymaloides*	Larvae and pupae	Denature ribosome, suppress the expression of enzymes and proteins crucial to ATP production causing disruption of the cell	AgNPs	15–30 nm	Spherical	UV-vis, FTIR, XRD, EDX, AFM		[114]
31.	*Pongamia pinnata*	Larvae	Not reported	AgNPs	10 to 80 nm	Spherical	UV-vis, XRD, FTIR, TEM	0.25–1 ppm	[115]
32.	*Delphinium denudatum*	Larvae II	DNA loses its replication ability and cellular proteins become inactivated on	AgNPs	85 nm	Spherical	UV-vis, XRD, SEM, FTIR	9.6 ppm	[116]
33.	*Bauhinia variegata*	Larvae III	Penetration through membrane to midgut epithelial membrane, the enzymes gets inactivated, and produce peroxide causing cell death	AgNPs	38 to 65 nm	Spherical, triangle, truncated triangles, and decahedral	UV-vis, XRD, SEM, FTIR,TEM, EDX	89.42 *μ*g/m L	[117]
34.	*Zornia diphylla*	Larvae III	Not reported	AgNPs	28 to 61 nm	Spheres, triangle, truncated triangles, and decahedral	UV-vis, XRD, SEM, FTIR,TEM, EDX	13.42 *μ*g/ml	[118]
35.	*Melia azedarach*	Larvae	Not reported	AgNPs	3 to 31 nm	Spherical	UV-vis, XRD,TEM,	23.82 ppm	[119]
36.	*Suaeda maritima*	Larvae I and pupae	Inhibit neurosecretory cells, causing shrinkage of internal cuticle, and/or can act directly on Epidermal cells responsible for the production of enzymes leading tanning and/or cuticular oxidation process	AgNPs	20 to 60 nm	Spherical	UV-vis, XRD, SEM, FTIR, EDX	8.668 to 17.975 ppm	[120]
37.	*Hedychium coronarium*	Larvae and pupae	Damaged midgut epithelium	AgNPs	9.54 nm to 49.0 nm	Spherical and oval	UV-vis, XRD, FTIR,TEM, EDX	24.2 ppm(I), 39.7 ppm(II), 52.7 ppm(III) 72.6 ppm(IV), 348.6 ppm	
38.	*Achyranthes aspera*	Larvae IV	Not reported	AgNPs	7 to 14 nm	Cuboidal and spherical	UV-vis, SEM, TEM, FTIR and XRD	8.92 mg/ml	[121]
39.	*Azadirachta indica*	Larvae III	Interfere with moulting and other physiological processes	AgNPs	41–60 nm	Spherical	UV-vis, XRD, SEM, FTIR,EDX	0.04 mg/l	[122]
40.	*Morinda citrifolia*	Larvae	Not reported	TiO_2_NPS	20.46–39.20 nm	Spherical, oval and triangle	UV- vis, XRD, SEM, FTIR,EDX	31.685 mg/L	[123]
41.	*Clausena dentata*	larvae	Denaturation of the sulfur-containing proteins or phosphorous- containing compound like DNA	SeNPs	46.32 nm to 78.88 nm	spherical	UV-vis, XRD, SEM, FTIR,EDX	104.13 mg/L	[124]
42.	*Hyptis suaveolens*	Larvae	Not reported	AgNPs	5–25 nm	Spherical, hexagonal, triangular and polyhedral	UV-vis, XRD, SEM, FTIR,TEM	10 mg/L	[125]
43.	*Chloroxylon swietenia*	Larvae	Not reported	AuNPs	18–37 nm	Spherical	UV-vis, XRD, FTIR, TEM, EDX, Zeta potential analyses	0.340 ppm	[126]
44.	*Ambrosia arborescens*	Larvae III	Bind macromolecules such as proteins and DNA, altering their structure	AgNPs	10–14 nm	Spherical	UV-vis,FTIR, TEM, SEM, EDX, AFM	0.43 ppm	[127]
45.	*Lobelia leschenaulti ana*	Larvae III	The disappearance of antenna and mouth brush, shrinkage in ventral area, loss of lateral hair, changes in structure of thorax, breakage of minutes of midgut, disappearance of anal gills, and brushes	ZnONps	15–46 nm	Spherical	UV-vis, XRD, FTIR, SEM, TEM	10 mg/L	[128]
46.	*Acacia caesia*	Larvae III, ova, adults	Midgut epithelial membrane damaged, enzymes were inactivated and generated peroxides leading to cell death	AgNPs	20 to 46 nm	Spherical	UV-vis, XRD, FTIR, EDX, SEM, TEM, AFM	11.32 *μ*g/ml for larvae, 75 *μ*g/m for ova, 20.94 *μ*g/ml for adults	[129]
47.	*Melia azedarach*	Larvae III	Interfere with intracellular cell signaling, bounds with sulfur contain proteins	Pd NPs	10 to 20 nm	Spherical	UV-vis, FTIR, XRD, TEM		[111]
48.	*Azadirachta indica*	Larvae III and IV	Increase ROS and other radicals production causing apoptosis via phosphatidyl serine externalization, DNA, nuclear fragmentation, activation of meta- caspases, mitochondrial dysfunction	AgNPs	35–60 nm	Spherical	UV-vis,SEM, EDX, TEM, FTIR, XRD, DLS	10.92 mg/L (III) 11.88 mg/L(IV)	[130]
49.	*Artocarpus heterophyllu s*	Larvae	Not reported	CuNPs	132 nm	Asymmetrical dispersed	UV-vis, XRD, FTIR,SEM	3.85, 4.24, 4.66 and 5.08 mg/ml	[131]
50.	*Morinda tinctoria*	Larvae III	Denature sulfur- containing proteins or phosphorous containing compound like DNA, causing in denaturation of organelles and enzymes	AgNPs	60–95 nm	Spherical	UV-vis, AFM, FTIR	3.631 ppm	[132]
51.	*Euphorbia milii*	Larvae II, IV	Not reported	AgNPs	208 nm	Spherical	UV-vis, SEM, EDX, XRD,FTIR, particle size, and zeta potential analysis	281.28 ± 23.30 and 178.97 ± 37.82 ppm	[133]
52.	*Mukia maderaspatana*	Larvae	Denature sulfur- containing proteins or phosphorous containing compound like DNA	AgNPs	13–34 nm	Spherical	UV-vis, XRD, FTIR, ART, SEM,	0.506; 1.082, 0.392; 0.870 ppm	[134]
53.	*Cassia fistula*	Larvae and pupae	Disturbed protein mechanism	AgNPs	148–938 nm	Spherical	FTIR, TEM, SEM, UV- vis, XRD	51.3, 47.1, 56.0, 78.0 and 519.3 mg/L	[135]
54.	*Chrysanthe mum* sp.	Larvae	Interference with the process of dissociation and other physiological processes	AgNPs	40–100 nm	Clustered and irregular shapes	UV-vis, FTIR, SEM	12.754 ppm	[136]
55.	*Carissa spinarum*	Larvae III	Not reported	AgNPs	40–100 nm	Cubic and spherical	FTIR, SEM, UV-vis, XRD, TEM	9.01 *μ*g/ml	[137]
56.	*Nicandra physalodes*	Larvae III	Interfere with molting and other physiological processes	AgNPs	5–35 nm	Cubic and spherical	UV-vis, XRD, FTIR, SEM, TEM	13.61 *µ*g/ml	[138]
57.	*Clerodendrum chinense*	Larvae III	Not reported	AgNPs	25–30 nm	Irregular, Spherical or with Cubic structures	UV-vis, SEM, TEM,EDX, FTIR	11.10 *µ*g/ml	[139]
58.	*Calotropis gigantea*	Larvae and pupae	Not reported	AgNPs	20–35 nm	Clustered and irregular	UV-vis, SEM, EDX, FTIR	24.33 ppm, 34.01 ppm, 51.92 ppm, 63.38 ppm and 83.88 ppm	[140]
59.	*Tagetes* sp.	Larvae IV	Not reported	CdNps		Roughly spherical	UV-vis, SEM, FTIR and fluorescence	10 ppm	[141]
60.	*Cleistanthus collinus*	Larvae	Inhibit neurosecretory cells and gut enzyme of larvae, toxic effect on epidermal cells	AgNPs	66.27 to 75.09 nm	Triangular and pentagonal	UV-vis, FTIR, XRD, SEM, EDX	20 mg/l	[142]
61.	*Strychnos nux-vomica*	Larvae	Inhibitory influence on neurosecretory cells and gut enzyme of larvae, toxic efficacy on epidermal cells	AgNPs	54.45 to 60.84 nm	Irregular, spherical and round	UV-vis, FTIR, XRD, SEM, EDX	25 mg/l	[142]
62.	*Tridax procumbens*	Larvae	Not reported	CuONps	16 nm		XRD, FTIR, SEM, EDX, UV-vis, and fluorescence spectroscopy	4.209 mg/L	[143]
63.	*Rhizophora mucronata*	Larvae III	Denaturation of the sulfur-containing proteins or phosphorous containing compound like DNA	AgNPs	60–95 nm	Spherical	UV-vis, XRD, FTIR, and AFM analysis	0.585 mg/L	[144]
64.	*Belosynapsis kewensis*	Larvae IV	Not reported	AgNPs	10 to 28 nm	Spherical	UV-vis, FTIR,TEM, and XRD	84.2 ppm	[145]
65.	*Cynodon dactylon*	Larvae	Bio uptake and toxicity	AgNPs	14 nm	Spherical	UV-vis, XRD, TEM	2.50, 2.78, 3.02, 3.05 *μ*g/mL	[146]
66.	*Sida acuta*	Adults	Interfere with molting and other physiological processes.	AgNPs	5–35 nm	Spherical	UV-vis, SEM, TEM, FTIR, EDX	35.12 *μ*g/mL	[147]
67.	*Mussaenda glabra*	Larvae	Not reported	AgNPs	15 to 25 nm	Spheres, Triangle, truncated Triangles and decahedral	UV-vis, XRD, FTIR, SEM, TEM	17–19 *μ*g/mL	[147]
68.	*Psychotria nilgiriensis*	Ova, larvae, pupae, adults	Not reported	AgNPs	40–60 nm	Spherical and cubic	UV-vis, SEM, FTIR, EDX	20.26, 24.08, 29.37, 35.33 and 43.12 *µ*g/ml	[148]
69.	*Berberis tinctoria*	Larvae and pupae	Interfere with molting and other physiological processes	AgNPs	65–70 nm	Spherical	UV-vis, XRD, SEM	4.97 ppm (I instar), 5.97 ppm (II), 7.60 ppm (III), 9.65 ppm (IV), and 14.87 ppm (pupa)	[149]
70.	*Derris trifoliata*	Larvae III and IV	Binding to DNA and enzymes and impairs cellular metabolism	AgNPs	18–50 nm	Spherical and cubic	UV-vis, FTIR, SEM, EDX, XRD, TEM	12.11 mg/l (III), 17.76 mg/l (IV)	[150]
71.	*Cassia roxburghii*	Larvae III	Not reported	Ag NPs	57 to 95 nm	Orbicular, trigonal, truncated triangles, and decahedral morphologies	UV-vis, FTIR, SEM, EDX, XRD.	31.27 and 48.81 *μ*g/mL	[151]
72.	*Artemisia nilagirica*	Larvae and pupae	Damage midgut epithelial membrane, inactivate enzymes and generate peroxide leading to cell death	AgNPs	6.723 nm	Spherical to irregular	UV-vis, FTIR, SEM, XRD		[152]
73.	*Scadoxus multiflorus*	Larvae and ova	Affect the epithelial cell/midgut or cortex, lateral hair loss, deformation in gills as well as brushes	ZnO NPs	31 ± 2 nm	Irregular spherical	UV-vis, FTIR, SEM, EDX, XRD	34.04 ppm and 32.73 ppm	[153]
74.	*Pergularia daemia*	Larvae	Not reported	AgNPs	44 to 255 nm	Spherical	UV-vis, TEM, particle size and zeta potential analysis	9.90, 11.13, 12.40, 12.95 ppm	[154]
75.	*Ipomoea batatas*	Larvae	DNA structure deformation, and generation of excessive reactive oxygen species.	AgNPs	20–50 nm	Orbicular	UV-vis, FTIR, SEM, EDX, XRD	15.657 *μ*g/mL	[155]
76.	*Annona squamosa*	Larvae	Not reported	AgNPs		Spherical and cluster shaped	UV-vis, XRD, FTIR, SEM	7.52, 8.34, 9.06, 9.15 *μ*g/mL	[156]
77.	*Achyranthes aspera*	Larvae IV	Reduce ATP synthesis, ion exchange, reduce membrane permeability causing cell death	AgNPs	1–30 nm	Three dimensional cuboid	UV-vis, FTIR, SEM, TEM, EDX, XRD	26.693 *μ*g/mL	[157]
78.	*Habenaria plantaginea*	Larvae	Not reported	AgNPs	0.1 to 29 nm	Polydispersed and spherical	UV-vis, AFM, FTIR, SEM, TEM, XRD	13.38 *μ*g/ml	[158]
79.	*Rubus ellipticus*	Larvae	Decrease membrane permeability, disturb proton motive process, Cellular function is disrupted	AgNPs	<30 nm	Spherical	UV-vis, XRD, FTIR, SEM, TEM, EDX	13.83 *μ*g/mL	[159]
80.	*Menyanthes trifoliata*	Adults	Detiriorated midgut	AgNPs	10 to 50 nm	Orbicular, Trigonal, pentagonal, hexagonal	UV-vis, XRD, FTIR, SEM, EDX	14.99 *μ*g/mL	[160]
81.	*Manihot esculenta*	Larvae III	Not reported	AgNPs		Spherical and aggregates	UV-vis, XRD, FESEM, and HRTEM	4.53 mg/mL	[161]
82.	*Couroupita guianensis*	Larvae IV	Not reported	AgNPs	10–45 nm	Spherical	UV-vis, XRD, FTIR, TEM	2.1 ppm	[162]
83.	*Couroupita guianensis*	Larvae IV	Not reported	AgNPs	5–15 nm	Orbicular	UV-vis, XRD, FTIR, TEM	2.09 ppm	[162]
84.	*Trichoderma atroviride*	Larvae	Not reported	AgNPs	14.01–21.02 nm	Hexagonal (diamond shape)	UV-vis, confocal laser microscopy (CLSM),	1 ppm, 2 pp m, 3.12 ppm, 6.30 ppm	[163]
85.	*Hedyotis puberula*	Larvae and ova	Not reported	AgNPs	10–16 nm	Mostly spherical, a few nanorods, hexagonal and polygonal nanoprisms	UV-vis, FTIR, XRD, AFM, SEM, TEM, EDX and DLS analysis	18.05 *µ*g/ml (larvae) 100 *µ*g/ml (ova)	[164]
86.	*Carica papaya*	Larvae II and III	Inhibit AChE, GABA- gated chloride ion channel, disruptna, K ion exchange, cyt-P450, hormones, osmotic pressure and ionic balance. cause mitotic poisoning, inhibit cholinergic system, neuromuscular coordination	AgNPs	12 ± 6 nm	Spherical	FTIR, GCMS	1.46 (II) 1.76 ppm (III)	[73]
87.	*Syzygium cumini*	Larvae	Not reported	AgNPs	50 nm	Spherical, round, triangular, and Hexagonal	UV-vis, FTIR, EDX, XRD, SEM	16.45 *µ*g/ml	[165]

NPs: nanoparticles; X-ray diffraction (XRD); Fourier transform infrared (FTIR); scanning electron microscope (SEM); energy dispersive X-ray analysis (EDX); UV-visible spectroscopy (UV-vis); field emission scanning electron microscope (FESEM); high resolution transmission electron microscopy (HRTEM); transmission electron microscopy (TEM); dynamic light scattering (DLS).

**Table 2:** Effect of NPs on the efficiency of predators of dengue vector.

S.No	Predator	Plant used	Nanoparticles (NPs)	Salt used (as a precursor)	Efficiency		Reference
					Before	After	
1	*Mesocyclo ps aspericornis*	*Cymbopogon citratus*	AuNPs	HAuCl_4_	56%	77.30%	[113]
2	*Megacyclo ps formosanus*	*Hedychium coronarium*	AgNPs	AgNO_3_	7.22, 5.88, 1.28, and 0.28 larvae	8.11, 6.88, 1.95, 1.06 larva/day	[168]
3	*Poecilia sphenops*	*Psychotria nilgiriensis*	AgNPs	AgNO_3_	65% (larva I), 49.62% (larva II)	92.25% (larva I), 76.50% (Larva II)	[148]
4	*Gambusia affinis*	*Mimusops elengi*	AgNPs	AgNO_3_	81.7% (larvae III)	88.60%	[169]
5	*Poecilia reticulata*	*Sonneratia alba*	AgNPs	AgNO_3_	6.5, 4.8, 3.8, 2.6 larvae/day	8.2, 6.4, 5.0, 3.9 larvae/day	[66]
6	*Oryzias melastigma*	*Chenopodium ambrosioides*	AgNPs	AgNO_3_	65.5 (II) and 59.0% (III)	91.0 (II) and 85.5% (III)	[170]

## Abbreviations

NPs: Nanoparticles
DENV-5: Dengue virus fifth serotype
DSS: Dengue shock syndrome
DHF: Dengue hemorrhagic fever
ADM: Antibody mediated disease enhancement
NS: Non-structural proteins
AgNPs: silver nanoparticles
AChE: Acetylcholinesterase
XRD: X-ray diffraction
FTIR: Fourier transform infrared
SEM: Scanning electron microscope
EDX: Energy dispersive X-ray analysis
UV-vis: UV-visible spectroscopy
FESEM: Field emission scanning electron microscope
HRTEM: High resolution transmission electron microscopy
TEM: Transmission electron microscopy
DLS: Dynamic light scattering.

## References

[1] Jones I. J., Sokolow S. H., De Leo G. A. (2022). Three reasons why expanded use of natural enemy solutions may offer sustainable control of human infections. *People and Nature*.

[2] Ayaz M., Ullah F., Sadiq A. (2019). Synergistic interactions of phytochemicals with antimicrobial agents: potential strategy to counteract drug resistance. *Chemico-Biological Interactions*.

[3] Ayaz M., Subhan F., Sadiq A., Ullah F., Ahmed J., Sewell R. D. E. (2017). Cellular efflux transporters and the potential role of natural products in combating efflux mediated drug resistance. *Frontiers in Bioscience*.

[4] Ayaz M., Nawaz A., Ahmad S. (2022). Underlying anticancer mechanisms and synergistic combinations of phytochemicals with cancer chemotherapeutics: potential benefits and risks. *Journal of Food Quality*.

[5] Mubemba B., Mburu M. M., Changula K. (2022). Current knowledge of vector-borne zoonotic pathogens in Zambia: a clarion call to scaling-up “one health” research in the wake of emerging and re-emerging infectious diseases. *PLoS Neglected Tropical Diseases*.

[6] Flores H. A., O’Neill S. L. (2018). Controlling vector-borne diseases by releasing modified mosquitoes. *Nature Reviews Microbiology*.

[7] Weaver S. C., Forrester N. L., Liu J., Vasilakis N. (2021). Population bottlenecks and founder effects: implications for mosquito-borne arboviral emergence. *Nature Reviews Microbiology*.

[8] Guzman M. G., Gubler D. J., Izquierdo A., Martinez E., Halstead S. B. (2016). Dengue infection. *Nature Reviews Disease Primers*.

[9] Ferreira-de-Lima V. H., Lima-Camara T. N. (2018). Natural vertical transmission of dengue virus in *Aedes aegypti* and *Aedes albopictus*: a systematic review. *Parasites & Vectors*.

[10] Bowman L. R., Donegan S., McCall P. J. (2016). Is dengue vector control deficient in effectiveness or evidence?: systematic review and meta-analysis. *PLoS Neglected Tropical Diseases*.

[11] Altassan K. K., Morin C., Shocket M. S., Ebi K., Hess J. (2019). Dengue fever in Saudi Arabia: a review of environmental and population factors impacting emergence and spread. *Travel Medicine and Infectious Disease*.

[12] Perera R., Kuhn R. J. (2008). Structural proteomics of dengue virus. *Current Opinion in Microbiology*.

[13] Joob B., Wiwanikit V. (2017). Dengue fever in Saudi Arabia. *Saudi Medical Journal*.

[14] Tahir ul Qamar M., Maryam A., Muneer I. (2019). Computational screening of medicinal plant phytochemicals to discover potent pan-serotype inhibitors against dengue virus. *Scientific Reports*.

[15] World Health Organization (2017). Dengue vaccine: WHO position paper, July 2016-recommendations. *Vaccine*.

[16] Paz-Bailey G., Adams L., Wong J. M. (2021). Dengue vaccine: recommendations of the advisory committee on immunization practices, United States, 2021. *Morbidity and Mortality Weekly Report Recommendations and Reports*.

[17] Flasche S., Jit M., Rodríguez-Barraquer I. (2016). The long-term safety, public health impact, and cost-effectiveness of routine vaccination with a recombinant, live-attenuated dengue vaccine (Dengvaxia): a model comparison study. *PLoS Medicine*.

[18] Jena N., Bal C., Sharon A. (2019). Plant and marine products: a promising hope in the search of therapeutics against dengue. *Discovery and Development of Therapeutics from Natural Products against Neglected Tropical Diseases*.

[19] Sujitha V., Murugan K., Paulpandi M. (2015). Green-synthesized silver nanoparticles as a novel control tool against dengue virus (DEN-2) and its primary vector *Aedes aegypti*. *Parasitology Research*.

[20] Borkow G., Lapidot A. (2005). Multi-targeting the entrance door to block HIV-1. *Current Drug Targets: Infectious Disorders*.

[21] Muthukumaran U., Govindarajan M., Rajeswary M. (2015). Mosquito larvicidal potential of silver nanoparticles synthesized using Chomelia asiatica (Rubiaceae) against *Anopheles stephensi*, *Aedes aegypti*, and *Culex quinquefasciatus* (Diptera: Culicidae). *Parasitology Research*.

[22] Benelli G., Murugan K., Panneerselvam C., Madhiyazhagan P., Conti B., Nicoletti M. (2015). Old ingredients for a new recipe? Neem cake, a low-cost botanical by-product in the fight against mosquito-borne diseases. *Parasitology Research*.

[23] Pavela R. (2009). Larvicidal property of essential oils against *Culex quinquefasciatus* Say (Diptera: Culicidae). *Industrial Crops and Products*.

[24] Hemingway J., Ranson H. (2000). Insecticide resistance in insect vectors of human disease. *Annual Review of Entomology*.

[25] Amer A., Mehlhorn H. (2006). Larvicidal effects of various essential oils against Aedes, Anopheles, and Culex larvae (Diptera, Culicidae). *Parasitology Research*.

[26] Millar J. G., Chaney J. D., Mulla M. S. (1992). Identification of oviposition attractants for *Culex quinquefasciatus* from fermented Bermuda grass infusions. *Journal of the American Mosquito Control Association*.

[27] Olagbemiro T. O., Birkett M. A., Mordue Lunt A. J., Pickett J. A. (1999). Production of (5 R, 6 S)-6-acetoxy-5-hexadecanolide, the mosquito oviposition pheromone, from the seed oil of the summer cypress plant, Kochia scoparia (Chenopodiaceae). *Journal of Agricultural and Food Chemistry*.

[28] Geetha I., Paily K., Padmanaban V., Balaraman K. (2003). Oviposition response of the mosquito, *Culex quinquefasciatus* to the secondary metabolite (s) of the fungus, Trichoderma viride. *Memorias do Instituto Oswaldo Cruz*.

[29] Benelli G., Rajeswary M., Govindarajan M. (2018). Towards green oviposition deterrents? Effectiveness of Syzygium lanceolatum (Myrtaceae) essential oil against six mosquito vectors and impact on four aquatic biological control agents. *Environmental Science and Pollution Research*.

[30] Bian G., Xu Y., Lu P., Xie Y., Xi Z. (2010). The endosymbiotic bacterium Wolbachia induces resistance to dengue virus in *Aedes aegypti*. *PLoS Pathogens*.

[31] Moreira L. A., Iturbe-Ormaetxe I., Jeffery J. A. (2009). A Wolbachia symbiont in *Aedes aegypti* limits infection with dengue, Chikungunya, and Plasmodium. *Cell*.

[32] Chittaranjan Patra I. A., Ayaz M., Khalil A. T., Mukherjee S., Ovais M. (2021). *Biogenic Nanoparticles for Cancer Theranostics*.

[33] Ovais M., Khalil A., Ayaz M., Ahmad I., Nethi S., Mukherjee S. (2018). Biosynthesis of metal nanoparticles via microbial enzymes: a mechanistic approach. *International Journal of Molecular Sciences*.

[34] Arul K. T., Manikandan E., Ladchumananandasivam R. (2019). Polymer-based calcium phosphate scaffolds for tissue engineering applications. *Nanoarchitectonics in Biomedicine*.

[35] Khalil A. T., Ovais M., Iqbal J. (2021). Microbes-mediated synthesis strategies of metal nanoparticles and their potential role in cancer therapeutics. *Seminars in Cancer Biology*.

[36] Khalil A. T., Iqbal J., Shah A. (2021). The bio–nano interface as an emerging trend in assembling multi-functional metal nanoparticles. *SPR Nanoscience*.

[37] Hassan D., Khalil A. T., Solangi A. R., El‐Mallul A., Shinwari Z. K., Maaza M. (2019). Physiochemical properties and novel biological applications of Callistemon viminalis‐mediated *α*‐Cr_2_O_3_ nanoparticles. *Applied Organometallic Chemistry*.

[38] Ahmad H., Venugopal K., Bhat A. H. (2020). Enhanced biosynthesis synthesis of copper oxide nanoparticles (CuO-NPs) for their antifungal activity toxicity against major phyto-pathogens of apple orchards. *Pharmaceutical Research*.

[39] Maaza M., Ngom B., Achouri M., Manikandan K. (2015). Functional nanostructured oxides. *Vacuum*.

[40] Ovais M., Ahmad I., Khalil A. T. (2018). Wound healing applications of biogenic colloidal silver and gold nanoparticles: recent trends and future prospects. *Applied Microbiology and Biotechnology*.

[41] Mwakikunga B. W., Forbes A., Sideras-Haddad E., Scriba M., Manikandan E. (2010). Self assembly and properties of C: WO3 nano-platelets and C: VO_2_/V_2_O_5_ triangular capsules produced by laser solution photolysis. *Nanoscale Research Letters*.

[42] Mohamed H. E. A., Afridi S., Khalil A. T. (2019). Phytosynthesis of BiVO_4_ nanorods using Hyphaene thebaica for diverse biomedical applications. *AMB Express*.

[43] Ayaz M., Ali T., Sadiq A., Ullah F., Naseer M. I. (2022). Current trends in medicinal plant research and neurodegenerative disorders. *Frontiers in Pharmacology*.

[44] Ayaz M., Sadiq A., Mosa O. F. (2022). Antioxidant, enzyme inhibitory, and molecular docking approaches to the antidiabetic potentials of bioactive compounds from persicaria hydropiper L. *Evidence-Based Complementary and Alternative Medicine*.

[45] Akhtar M. F., Mehal M. O., Saleem A. (2022). Attenuating effect of prosopis cineraria against paraquat-induced toxicity in prepubertal mice, *Mus musculus*. *Environmental Science and Pollution Research*.

[46] Ahmad S., Ullah F., Ayaz M., Ahmad A., Sadiq A., Mohani S. N.-U.-H. (2019). Nutritional and medicinal aspects of Rumex hastatus D. Don along with in vitro anti-diabetic activity. *International Journal of Food Properties*.

[47] Mahnashi M. H., Alqahtani Y. S., Alyami B. A. (2021). Cytotoxicity, anti-angiogenic, anti-tumor and molecular docking studies on phytochemicals isolated from Polygonum hydropiper L. *BMC Complementary Medicine and Therapies*.

[48] Ullah I., Subhan F., Ayaz M. (2015). Anti-emetic mechanisms of zingiber officinale against cisplatin induced emesis in the pigeon; behavioral and neurochemical correlates. *BMC Complementary and Alternative Medicine*.

[49] Ghufran M., Rehman A. U., Shah M., Ayaz M., Ng H. L., Wadood A. (2020). In-silico design of peptide inhibitors of K-Ras target in cancer disease. *Journal of Biomolecular Structure and Dynamics*.

[50] Saleem U., Akhtar R., Anwar F. (2021). Neuroprotective potential of malva neglecta is mediated via down-regulation of cholinesterase and modulation of oxidative stress markers. *Metabolic Brain Disease*.

[51] Qamar T., Mumtaz A., Ashfaq U. A. (2014). Computer aided screening of phytochemicals from garcinia against the dengue NS2B/NS3 protease. *Bioinformation*.

[52] Martins L. L., Gilson L. L., Maynard M. T. (2004). Virtual teams: what do we know and where do we go from here?. *Journal of Management*.

[53] Sood R., Raut R., Tyagi P. (2015). Cissampelos pareira Linn: natural source of potent antiviral activity against all four dengue virus serotypes. *PLoS Neglected Tropical Diseases*.

[54] Abd Kadir S. L., Yaakob H., Mohamed Zulkifli R. (2013). Potential anti-dengue medicinal plants: a review. *Journal of Natural Medicines*.

[55] Mukim M., Kabra A., Hano C. (2022). Rivea hypocrateriformis (desr.) choisy: an overview of its ethnomedicinal uses, phytochemistry, and biological activities and prospective research directions. *Journal of Chemistry*.

[56] Nasar M. Q., Khalil A. T., Ali M., Shah M., Ayaz M., Shinwari Z. K. (2019). Phytochemical analysis, Ephedra Procera CA Mey. Mediated green synthesis of silver nanoparticles, their cytotoxic and antimicrobial potentials. *Medicina*.

[57] Qasim Nasar M., Zohra T., Khalil A. T. (2019). Seripheidium quettense mediated green synthesis of biogenic silver nanoparticles and their theranostic applications. *Green Chemistry Letters and Reviews*.

[58] Nasar M. Q., Shah M., Khalil A. T. (2022). Ephedra intermedia mediated synthesis of biogenic silver nanoparticles and their antimicrobial, cytotoxic and hemocompatability evaluations. *Inorganic Chemistry Communications*.

[59] Ovais M., Khalil A. T., Ayaz M., Ahmad I. (2019). Biosynthesized metallic nanoparticles as emerging cancer theranostics agents. *Nanotheranostics*.

[60] Ovais M., Khalil A. T., Ayaz M., Ahmad I. (2020). Metal oxide nanoparticles and plants. *Phytonanotechnology*.

[61] Sani A., Hassan D., Khalil A. T. (2021). Floral extracts-mediated green synthesis of NiO nanoparticles and their diverse pharmacological evaluations. *Journal of Biomolecular Structure and Dynamics*.

[62] Khalil A. T., Khan M. D., Razzaque S. (2021). Single precursor-based synthesis of transition metal sulfide nanoparticles and evaluation of their antimicrobial, antioxidant and cytotoxic potentials. *Applied Nanoscience*.

[63] Khalil A. T., Ayaz M., Ovais M. (2018). In vitro cholinesterase enzymes inhibitory potential and in silico molecular docking studies of biogenic metal oxides nanoparticles. *Inorganic and Nano-Metal Chemistry*.

[64] Ayaz M., Ovais M., Ahmad I., Sadiq A., Khalil A. T., Ullah F. (2020). Biosynthesized metal nanoparticles as potential Alzheimer’s disease therapeutics. *Metal Nanoparticles for Drug Delivery and Diagnostic Applications*.

[65] Murugan K., Dinesh D., Paulpandi M. (2015). Nanoparticles in the fight against mosquito-borne diseases: bioactivity of Bruguiera cylindrica-synthesized nanoparticles against dengue virus DEN-2 (in vitro) and its mosquito vector *Aedes aegypti* (Diptera: Culicidae). *Parasitology Research*.

[66] Murugan K., Dinesh D., Paulpandi M. (2017). Mangrove helps: sonneratia alba-synthesized silver nanoparticles magnify guppy fish predation against *Aedes aegypti* young instars and down-regulate the expression of envelope (E) gene in dengue virus (serotype DEN-2). *Journal of Cluster Science*.

[67] Murugan K., Aruna P., Panneerselvam C. (2016). Fighting arboviral diseases: low toxicity on mammalian cells, dengue growth inhibition (in vitro), and mosquitocidal activity of Centroceras clavulatum-synthesized silver nanoparticles. *Parasitology Research*.

[68] Ntoumba A. A., Meva F. E., Ekoko W. E. (2020). Biogenic synthesis of silver nanoparticles using guava (Psidium guajava) leaf extract and its larvicidal action against *Anopheles gambiae*. *Journal of Biomaterials and Nanobiotechnology*.

[69] Shah A., Manikandan E., Basheer Ahamed M., Ahmad Mir D., Ahmad Mir S. (2014). Antibacterial and blue shift investigations in sol-gel synthesized Cr_*x*_Zn_1−*x*_O nanostructures. *Journal of Luminescence*.

[70] Schoelitsz B., Meerburg B. G., Takken W. (2019). Influence of the public’s perception, attitudes, and knowledge on the implementation of integrated pest management for household insect pests. *Entomologia Experimentalis et Applicata*.

[71] Singh N. S., Sharma R., Parween T., Patanjali P. (2018). Pesticide contamination and human health risk factor. *Modern Age Environmental Problems and Their Remediation*.

[72] Shahzad K., Manzoor F. (2019). Nanoformulations and their mode of action in insects: a review of biological interactions. *Drug and Chemical Toxicology*.

[73] Chandrasekaran R., Seetharaman P., Krishnan M., Gnanasekar S., Sivaperumal S. (2018). Carica papaya (Papaya) latex: a new paradigm to combat against dengue and filariasis vectors *Aedes aegypti* and *Culex quinquefasciatus* (Diptera: Culicidae). *3 Biotech*.

[74] Benelli G. (2018). Mode of action of nanoparticles against insects. *Environmental Science and Pollution Research*.

[75] López M., Pascual-Villalobos M. (2010). Mode of inhibition of acetylcholinesterase by monoterpenoids and implications for pest control. *Industrial Crops and Products*.

[76] Mir N. T., Saleem U., Anwar F. (2019). Lawsonia Inermis markedly improves cognitive functions in animal models and modulate oxidative stress markers in the brain. *Medicina*.

[77] Ovais M., Zia N., Ahmad I. (2018). Phyto-therapeutic and nanomedicinal approaches to cure Alzheimer’s disease: present status and future opportunities. *Frontiers in Aging Neuroscience*.

[78] Helvig C., Koener J. F., Unnithan G. C., Feyereisen R. (2004). CYP15A1, the cytochrome P450 that catalyzes epoxidation of methyl farnesoate to juvenile hormone III in cockroach corpora allata. *Proceedings of the National Academy of Sciences*.

[79] Rai M., Kon K., Ingle A., Duran N., Galdiero S., Galdiero M. (2014). Broad-spectrum bioactivities of silver nanoparticles: the emerging trends and future prospects. *Applied Microbiology and Biotechnology*.

[80] Benelli G. (2016). Plant-mediated biosynthesis of nanoparticles as an emerging tool against mosquitoes of medical and veterinary importance: a review. *Parasitology Research*.

[81] Jiang X., Miclăuş T., Wang L. (2015). Fast intracellular dissolution and persistent cellular uptake of silver nanoparticles in CHO-K1 cells: implication for cytotoxicity. *Nanotoxicology*.

[82] Handy R. D., Owen R., Valsami-Jones E. (2008). The ecotoxicology of nanoparticles and nanomaterials: current status, knowledge gaps, challenges, and future needs. *Ecotoxicology*.

[83] Scown T. M., Van Aerle R., Tyler C. R. (2010). Do engineered nanoparticles pose a significant threat to the aquatic environment?. *Critical Reviews in Toxicology*.

[84] Gomes T., Araújo O., Pereira R., Almeida A. C., Cravo A., Bebianno M. J. (2013). Genotoxicity of copper oxide and silver nanoparticles in the mussel *Mytilus galloprovincialis*. *Marine Environmental Research*.

[85] Canesi L., Ciacci C., Fabbri R., Marcomini A., Pojana G., Gallo G. (2012). Bivalve molluscs as a unique target group for nanoparticle toxicity. *Marine Environmental Research*.

[86] Suganya G., Karthi S., Shivakumar M. S. (2014). Larvicidal potential of silver nanoparticles synthesized from Leucas aspera leaf extracts against dengue vector *Aedes aegypti*. *Parasitology Research*.

[87] Veerakumar K., Govindarajan M. (2014). Adulticidal properties of synthesized silver nanoparticles using leaf extracts of Feronia elephantum (Rutaceae) against filariasis, malaria, and dengue vector mosquitoes. *Parasitology Research*.

[88] Santhosh S. B., Yuvarajan R., Natarajan D. (2015). Annona muricata leaf extract-mediated silver nanoparticles synthesis and its larvicidal potential against dengue, malaria and filariasis vector. *Parasitology Research*.

[89] Suresh U., Murugan K., Benelli G. (2015). Tackling the growing threat of dengue: Phyllanthus niruri-mediated synthesis of silver nanoparticles and their mosquitocidal properties against the dengue vector *Aedes aegypti* (Diptera: Culicidae). *Parasitology Research*.

[90] Kumar D., Kumar G., Agrawal V. (2018). Green synthesis of silver nanoparticles using Holarrhena antidysenterica (L.) Wall. bark extract and their larvicidal activity against dengue and filariasis vectors. *Parasitology Research*.

[91] Ramkumar G., Karthi S., Suganya R., Shivakumar M. S. (2016). Evaluation of silver nanoparticle toxicity of Coleus aromaticus leaf extracts and its larvicidal toxicity against dengue and filariasis vectors. *BioNanoScience*.

[92] Murugan K., Priyanka V., Dinesh D. (2015). Predation by Asian bullfrog tadpoles, *Hoplobatrachus tigerinus*, against the dengue vector, *Aedes aegypti*, in an aquatic environment treated with mosquitocidal nanoparticles. *Parasitology Research*.

[93] Kalimuthu K., Panneerselvam C., Chou C. (2017). Predatory efficiency of the copepod Megacyclops formosanus and toxic effect of the red alga Gracilaria firma-synthesized silver nanoparticles against the dengue vector *Aedes aegypti*. *Hydrobiologia*.

[94] Ashokan A. P., Paulpandi M., Dinesh D., Murugan K., Vadivalagan C., Benelli G. (2017). Toxicity on dengue mosquito vectors through Myristica fragrans-synthesized zinc oxide nanorods, and their cytotoxic effects on liver cancer cells (HepG2). *Journal of Cluster Science*.

[95] Banu A. N., Balasubramanian C. (2014). Myco-synthesis of silver nanoparticles using Beauveria bassiana against dengue vector, *Aedes aegypti* (Diptera: Culicidae). *Parasitology Research*.

[96] Benelli G., Govindarajan M. (2017). Green-synthesized mosquito oviposition attractants and ovicides: towards a nanoparticle-based “lure and kill” approach?. *Journal of Cluster Science*.

[97] Gomathi M., Prakasam A., Chandrasekaran R., Gurusubramaniam G., Revathi K., Rajeshkumar S. (2019). Assessment of silver nanoparticle from cocos nucifera (coconut) shell on dengue vector toxicity, detoxifying enzymatic activity and predatory response of aquatic organism. *Journal of Cluster Science*.

[98] Govindarajan M., Benelli G. (2017). A facile one-pot synthesis of eco-friendly nanoparticles using carissacarandas: ovicidal and larvicidal potential on malaria, dengue and filariasis mosquito vectors. *Journal of Cluster Science*.

[99] Kovendan K., Chandramohan B., Govindarajan M. (2018). Orchids as sources of novel nanoinsecticides? Efficacy of Bacillus sphaericus and Zeuxine gracilis-fabricated silver nanoparticles against dengue, malaria and filariasis mosquito vectors. *Journal of Cluster Science*.

[100] Mahyoub J. A., Aziz A. T., Panneerselvam C. (2017). Seagrasses as sources of mosquito nano-larvicides? toxicity and uptake of Halodule uninervis-biofabricated silver nanoparticles in dengue and Zika virus vector *Aedes aegypti*. *Journal of Cluster Science*.

[101] Thandapani K., Kathiravan M., Namasivayam E. (2018). Enhanced larvicidal, antibacterial, and photocatalytic efficacy of TiO_2_ nanohybrids green synthesized using the aqueous leaf extract of Parthenium hysterophorus. *Environmental Science and Pollution Research*.

[102] Veerakumar K., Govindarajan M., Rajeswary M. (2013). Green synthesis of silver nanoparticles using Sida acuta (Malvaceae) leaf extract against *Culex quinquefasciatus*, *Anopheles stephensi*, and *Aedes aegypti* (Diptera: Culicidae). *Parasitology Research*.

[103] Velu K., Elumalai D., Hemalatha P. (2015). Evaluation of silver nanoparticles toxicity of *Arachis hypogaea* peel extracts and its larvicidal activity against malaria and dengue vectors. *Environmental Science and Pollution Research*.

[104] Chandramohan B., Murugan K., Panneerselvam C. (2016). Characterization and mosquitocidal potential of neem cake-synthesized silver nanoparticles: genotoxicity and impact on predation efficiency of mosquito natural enemies. *Parasitology Research*.

[105] Veerakumar K., Govindarajan M., Rajeswary M., Muthukumaran U. (2014). Retracted article:Mosquito larvicidal properties of silver nanoparticles synthesized using Heliotropium indicum (Boraginaceae) against *Aedes aegypti*, *Anopheles stephensi*, and *Culex quinquefasciatus* (Diptera: Culicidae). *Parasitology Research*.

[106] Veerakumar K., Govindarajan M., Rajeswary M., Muthukumaran U. (2014). Low-cost and eco-friendly green synthesis of silver nanoparticles using Feronia elephantum (Rutaceae) against *Culex quinquefasciatus*, *Anopheles stephensi*, and *Aedes aegypti* (Diptera: Culicidae). *Parasitology Research*.

[107] Rajkumar R., Shivakumar M. S., Senthil Nathan S., Selvam K. (2018). Pharmacological and larvicidal potential of green synthesized silver nanoparticles using carmona retusa (vahl) masam leaf extract. *Journal of Cluster Science*.

[108] Patil C. D., Patil S. V., Borase H. P., Salunke B. K., Salunkhe R. B. (2012). Larvicidal activity of silver nanoparticles synthesized using Plumeria rubra plant latex against *Aedes aegypti* and *Anopheles stephensi*. *Parasitology Research*.

[109] Pavunraj M., Baskar K., Duraipandiyan V., Al-Dhabi N. A., Rajendran V., Benelli G. (2017). Toxicity of Ag nanoparticles synthesized using stearic acid from Catharanthus roseus leaf extract against Earias vittella and mosquito vectors (*Culex quinquefasciatus* and *Aedes aegypti*). *Journal of Cluster Science*.

[110] Govindarajan M., Rajeswary M., Veerakumar K., Muthukumaran U., Hoti S., Benelli G. (2016). Green synthesis and characterization of silver nanoparticles fabricated using Anisomeles indica: mosquitocidal potential against malaria, dengue and Japanese encephalitis vectors. *Experimental Parasitology*.

[111] Ishwarya R., Vaseeharan B., Kalyani S. (2018). Facile green synthesis of zinc oxide nanoparticles using Ulva lactuca seaweed extract and evaluation of their photocatalytic, antibiofilm and insecticidal activity. *Journal of Photochemistry and Photobiology B: Biology*.

[112] Madhiyazhagan P., Murugan K., Kumar A. N. (2015). Sargassum muticum-synthesized silver nanoparticles: an effective control tool against mosquito vectors and bacterial pathogens. *Parasitology Research*.

[113] Murugan K., Benelli G., Panneerselvam C. (2015). Cymbopogon citratus-synthesized gold nanoparticles boost the predation efficiency of copepod Mesocyclops aspericornis against malaria and dengue mosquitoes. *Experimental Parasitology*.

[114] Sundaravadivelan C., Nalini Padmanabhan M., Sivaprasath P., Kishmu L. (2013). Biosynthesized silver nanoparticles from Pedilanthus tithymaloides leaf extract with anti-developmental activity against larval instars of *Aedes aegypti* L.(Diptera; Culicidae). *Parasitology Research*.

[115] Naik B. R., Gowreeswari G. S., Singh Y., Satyavathi R., Daravath S. S., Reddy P. R. (2014). Bio-Synthesis of Silver Nanoparticles from Leaf Extract of Pongamia pinnata as an Effective Larvicide on Dengue Vector *Aedes albopictus* (Skuse) (Diptera: Culicidae). *Advances in Entomology*.

[116] Suresh G., Gunasekar P. H., Kokila D. (2014). Green synthesis of silver nanoparticles using Delphinium denudatum root extract exhibits antibacterial and mosquito larvicidal activities. *Spectrochimica Acta Part A: Molecular and Biomolecular Spectroscopy*.

[117] Govindarajan M., Rajeswary M., Veerakumar K. (2016). Retracted article: novel synthesis of silver nanoparticles using Bauhinia variegata: a recent eco-friendly approach for mosquito control. *Parasitology Research*.

[118] Govindarajan M., Rajeswary M., Muthukumaran U., Hoti S., Khater H. F., Benelli G. (2016). Single-step biosynthesis and characterization of silver nanoparticles using Zornia diphylla leaves: a potent eco-friendly tool against malaria and arbovirus vectors. *Journal of Photochemistry and Photobiology B: Biology*.

[119] Ramanibai R., Velayutham K. (2015). Bioactive compound synthesis of Ag nanoparticles from leaves of Melia azedarach and its control for mosquito larvae. *Research in Veterinary Science*.

[120] Suresh U., Murugan K., Panneerselvam C. (2018). Suaeda maritima-based herbal coils and green nanoparticles as potential biopesticides against the dengue vector *Aedes aegypti* and the tobacco cutworm Spodoptera litura. *Physiological and Molecular Plant Pathology*.

[121] Elumalai D., Kaleena P., Ashok K., Suresh A., Hemavathi M. (2016). Green synthesis of silver nanoparticle using *Achyranthes aspera* and its larvicidal activity against three major mosquito vectors. *Engineering in Agriculture, Environment and Food*.

[122] Poopathi S., De Britto L. J., Praba V. L., Mani C., Praveen M. (2015). Synthesis of silver nanoparticles from Azadirachta indica—a most effective method for mosquito control. *Environmental Science and Pollution Research*.

[123] Suman T. Y., Ravindranath R. R. S., Elumalai D. (2015). Larvicidal activity of titanium dioxide nanoparticles synthesized using *Morinda citrifolia* root extract against *Anopheles stephensi*, *Aedes aegypti* and *Culex quinquefasciatus* and its other effect on non-target fish. *Asian Pacific Journal of Tropical Disease*.

[124] Sowndarya P., Ramkumar G., Shivakumar M. S. (2017). Green synthesis of selenium nanoparticles conjugated Clausena dentata plant leaf extract and their insecticidal potential against mosquito vectors. *Artificial Cells, Nanomedicine, and Biotechnology*.

[125] Elumalai D., Hemavathi M., Deepaa C. V., Kaleena P. K. (2017). Evaluation of phytosynthesised silver nanoparticles from leaf extracts of Leucas aspera and Hyptis suaveolens and their larvicidal activity against malaria, dengue and filariasis vectors. *Parasite Epidemiology and Control*.

[126] Balasubramani G., Ramkumar R., Krishnaveni N. (2015). GC-MS analysis of bioactive components and synthesis of gold nanoparticle using Chloroxylon swietenia DC leaf extract and its larvicidal activity. *Journal of Photochemistry and Photobiology B: Biology*.

[127] Morejón B., Pilaquinga F., Domenech F., Ganchala D., Debut A., Neira M. (2018). Larvicidal activity of silver nanoparticles synthesized using extracts of ambrosia arborescens (asteraceae) to control *Aedes aegypti* L.(Diptera: Culicidae). *Journal of Nanotechnology*.

[128] Banumathi B., Vaseeharan B., Ishwarya R. (2017). Toxicity of herbal extracts used in ethno-veterinary medicine and green-encapsulated ZnO nanoparticles against *Aedes aegypti* and microbial pathogens. *Parasitology Research*.

[129] Benelli G., Kadaikunnan S., Alharbi N. S., Govindarajan M. (2018). Biophysical characterization of Acacia caesia-fabricated silver nanoparticles: effectiveness on mosquito vectors of public health relevance and impact on non-target aquatic biocontrol agents. *Environmental Science and Pollution Research*.

[130] Siddhardha B., Sairengpuii H., Jobina R. (2015). Biogenic synthesis of silver nanoparticles using aqueous floral extract of Azadirachta indica and its anti‐Candida and larvicidal activities. *Research Journal of Chemistry and Environment*.

[131] Sharon E. A., Velayutham K., Ramanibai R. (2018). Biosynthesis of copper nanoparticles using artocarpus heterophyllus against dengue vector *Aedes aegypti*. *International Journal of Life-Sciences Scientific Research ISSN*.

[132] Ramesh Kumar K., Nattuthurai G. P., Mariappan T. (2014). Biosynthesis of silver nanoparticles from Morinda tinctoria leaf extract and their larvicidal activity against *Aedes aegypti* Linnaeus 1762. *Journal of Nanomedicine & Nanotechnology*.

[133] Borase H., Patil C., Salunkhe R. (2014). Mosquito larvicidal and silver nanoparticles synthesis potential of plant latex. *Journal of Entomological and Acarological Research*.

[134] Chitra G., Balasubramani G., Ramkumar R., Sowmiya R., Perumal P. (2015). *Mukia maderaspatana* (Cucurbitaceae) extract-mediated synthesis of silver nanoparticles to control *Culex quinquefasciatus* and *Aedes aegypti* (Diptera: Culicidae). *Parasitology Research*.

[135] Fouad H., Hongjie L., Hosni D. (2018). Controlling *Aedes albopictus* and *Culex pipiens* pallens using silver nanoparticles synthesized from aqueous extract of Cassia fistula fruit pulp and its mode of action. *Artificial Cells, Nanomedicine, and Biotechnology*.

[136] Ghramh H. A., Al-Ghamdi K. M., Mahyoub J. A., Ibrahim E. H. (2018). Chrysanthemum extract and extract prepared silver nanoparticles as biocides to control *Aedes aegypti* (L.), the vector of dengue fever. *Journal of Asia-Pacific Entomology*.

[137] Govindarajan M., Nicoletti M., Benelli G. (2016). Bio-physical characterization of poly-dispersed silver nanocrystals fabricated using Carissa spinarum: a potent tool against mosquito vectors. *Journal of Cluster Science*.

[138] Govindarajan M., Khater H. F., Panneerselvam C., Benelli G. (2016). One-pot fabrication of silver nanocrystals using Nicandra physalodes: a novel route for mosquito vector control with moderate toxicity on non-target water bugs. *Research in Veterinary Science*.

[139] Govindarajan M., Rajeswary M., Hoti S. (2016). Clerodendrum chinense-mediated biofabrication of silver nanoparticles: mosquitocidal potential and acute toxicity against non-target aquatic organisms. *Journal of Asia-Pacific Entomology*.

[140] Priya S., Murugan K., Priya A., Dinesh D., Panneerselvam C., Devi G. D. (2014). Green synthesis of silver nanoparticles using calotropis gigantea and their potential mosquito larvicidal property. *Journal of Pure and Applied Zoology*.

[141] Hajra A., Dutta S., Mondal N. K. (2016). Mosquito larvicidal activity of cadmium nanoparticles synthesized from petal extracts of marigold (Tagetes sp.) and rose (Rosa sp.) flower. *Journal of Parasitic Diseases*.

[142] Jinu U., Rajakumaran S., Senthil-Nathan S., Geetha N., Venkatachalam P. (2018). Potential larvicidal activity of silver nanohybrids synthesized using leaf extracts of Cleistanthus collinus (Roxb.) Benth. ex Hook. f. and Strychnos nux-vomica L. nux-vomica against dengue, Chikungunya and Zika vectors. *Physiological and Molecular Plant Pathology*.

[143] Muthamil Selvan S., Vijai Anand K., Govindaraju K. (2018). Green synthesis of copper oxide nanoparticles and mosquito larvicidal activity against dengue, zika and chikungunya causing vector *Aedes aegypti*. *IET Nanobiotechnology*.

[144] Gnanadesigan M., Anand M., Ravikumar S. (2011). Biosynthesis of silver nanoparticles by using mangrove plant extract and their potential mosquito larvicidal property. *Asian Pacific Journal of Tropical Medicine*.

[145] Bhuvaneswari R., Xavier R. J., Arumugam M. (2016). Larvicidal property of green synthesized silver nanoparticles against vector mosquitoes (*Anopheles stephensi* and *Aedes aegypti*). *Journal of King Saud University Science*.

[146] Ramanibai R., Velayutham K. (2016). Synthesis of silver nanoparticles using 3,5-di-t-butyl-4-hydroxyanisole from Cynodon dactylon against *Aedes aegypti* and *Culex quinquefasciatus*. *Journal of Asia-Pacific Entomology*.

[147] Govindarajan K. V. M., Murugan K. (2014). Single-step novel biosynthesis of silver nanoparticles: a potent and eco-friendly mosquitocides. *Journal of Ecobiotechnology*.

[148] Kovendan K., Chandramohan B., Dinesh D. (2016). Green-synthesized silver nanoparticles using Psychotria nilgiriensis: toxicity against the dengue vector *Aedes aegypti* (Diptera: Culicidae) and impact on the predatory efficiency of the non-target organism *Poecilia sphenops* (Cyprinodontiformes: poeciliidae). *Journal of Asia-Pacific Entomology*.

[149] Kumar P. M., Murugan K., Madhiyazhagan P. (2016). Biosynthesis, characterization, and acute toxicity of Berberis tinctoria-fabricated silver nanoparticles against the Asian tiger mosquito, *Aedes albopictus*, and the mosquito predators *Toxorhynchites splendens* and Mesocyclops thermocyclopoides. *Parasitology Research*.

[150] Kumar V. A., Ammani K., Jobina R., Subhaswaraj P., Siddhardha B. (2017). Photo-induced and phytomediated synthesis of silver nanoparticles using *Derris trifoliata* leaf extract and its larvicidal activity against *Aedes aegypti*. *Journal of Photochemistry and Photobiology B: Biology*.

[151] Muthukumaran U., Govindarajan M., Rajeswary M. (2015). Green synthesis of silver nanoparticles from Cassia roxburghii—a most potent power for mosquito control. *Parasitology Research*.

[152] Nalini M., Lena M., Sumathi P., Sundaravadivelan C. (2017). Effect of phyto-synthesized silver nanoparticles on developmental stages of malaria vector, *Anopheles stephensi* and dengue vector, *Aedes aegypti*. *Egyptian Journal of Basic and Applied Sciences*.

[153] Al-Dhabi N., Valan Arasu M. (2018). Environmentally-friendly green approach for the production of zinc oxide nanoparticles and their anti-fungal, ovicidal, and larvicidal properties. *Nanomaterials*.

[154] Patil C. D., Borase H. P., Patil S. V., Salunkhe R. B., Salunke B. K. (2012). Larvicidal activity of silver nanoparticles synthesized using Pergularia daemia plant latex against *Aedes aegypti* and *Anopheles stephensi* and nontarget fish Poecillia reticulata. *Parasitology Research*.

[155] Pavithra Bharathi V., Ragavendran C., Murugan N., Natarajan D. (2017). Ipomoea batatas (Convolvulaceae)-mediated synthesis of silver nanoparticles for controlling mosquito vectors of *Aedes albopictus*, *Anopheles stephensi*, and *Culex quinquefasciatus* (Diptera: Culicidae). *Artificial Cells, Nanomedicine, and Biotechnology*.

[156] Velayutham K., Ramanibai R. (2016). Larvicidal activity of synthesized silver nanoparticles using isoamyl acetate identified in Annona squamosa leaves against *Aedes aegypti* and *Culex quinquefasciatus*. *The Journal of Basic & Applied Zoology*.

[157] Sharma A., Kumar S., Tripathi P. (2019). A facile and rapid method for green synthesis of *Achyranthes aspera* stem extract-mediated silver nano-composites with cidal potential against *Aedes aegypti* L. *Saudi Journal of Biological Sciences*.

[158] Aarthi C., Govindarajan M., Rajaraman P. (2018). Eco-friendly and cost-effective Ag nanocrystals fabricated using the leaf extract of Habenaria plantaginea: toxicity on six mosquito vectors and four non-target species. *Environmental Science and Pollution Research*.

[159] AlQahtani F. S., AlShebly M. M., Govindarajan M., Senthilmurugan S., Vijayan P., Benelli G. (2017). Green and facile biosynthesis of silver nanocomposites using the aqueous extract of Rubus ellipticus leaves: toxicity and oviposition deterrent activity against Zika virus, malaria and filariasis mosquito vectors. *Journal of Asia-Pacific Entomology*.

[160] Kamalakannan S., Ramar M., Arumugam P. (2018). Exploration of distinctive menyanthes trifoliata as generated green nanoparticles: report their lethal toxicity against aedes aegypti. *American Journal of Innovative Research and Applied Sciences*.

[161] Velayutham K., Ramanibai R., Umadevi M. (2016). Green synthesis of silver nanoparticles using Manihot esculenta leaves against *Aedes aegypti* and *Culex quinquefasciatus*. *The Journal of Basic & Applied Zoology*.

[162] Vimala R., Sathishkumar G., Sivaramakrishnan S. (2015). Optimization of reaction conditions to fabricate nano-silver using Couroupita guianensis Aubl.(leaf & fruit) and its enhanced larvicidal effect. *Spectrochimica Acta Part A: Molecular and Biomolecular Spectroscopy*.

[163] Singh G., Prakash S. (2015). Virulency of novel nanolarvicide from Trichoderma atroviride against *Aedes aegypti* (Linn.): a CLSM analysis. *Environmental Science and Pollution Research*.

[164] Thameem Azarudeen R. M. S., Govindarajan M., Amsath A. (2016). Size-controlled fabrication of silver nanoparticles using the Hedyotis puberula leaf extract: toxicity on mosquito vectors and impact on biological control agents. *RSC Advances*.

[165] Kanthammal S., Jebanesan A., Kovendan K., Subramaniam J., Vijay M. (2018). Novel insecticides of Syzygium cumini fabricated silver nanoparticles against filariasis, malaria, and dengue vector mosquitoes. *International Journal of Mosquito Research*.

[166] Chitra S., Jayaprakash K. (2013). Effect of mercury on blood components of fresh water edible fish Labeo rohita. *Hemoglobin*.

[167] Kalimuthu S., Se-Kwon K. (2013). Cell survival and apoptosis signaling as therapeutic target for cancer: marine bioactive compounds. *International Journal of Molecular Sciences*.

[168] Kalimuthu K., Panneerselvam C., Chou C. (2017). Control of dengue and Zika virus vector *Aedes aegypti* using the predatory copepod Megacyclops formosanus: synergy with Hedychium coronarium-synthesized silver nanoparticles and related histological changes in targeted mosquitoes. *Process Safety and Environmental Protection*.

[169] Subramaniam J., Murugan K., Panneerselvam C. (2015). Eco-friendly control of malaria and arbovirus vectors using the mosquitofish *Gambusia affinis* and ultra-low dosages of Mimusops elengi-synthesized silver nanoparticles: towards an integrative approach?. *Environmental Science and Pollution Research*.

[170] Subramaniam J., Murugan K., Jebanesan A. (2017). Do Chenopodium ambrosioides-synthesized silver nanoparticles impact Oryzias melastigma predation against *Aedes albopictus* larvae?. *Journal of Cluster Science*.

